# Impacts of Small-Scale Industrialized Swine Farming on Local Soil, Water and Crop Qualities in a Hilly Red Soil Region of Subtropical China

**DOI:** 10.3390/ijerph14121524

**Published:** 2017-12-06

**Authors:** Di Zhang, Xingxiang Wang, Zhigao Zhou

**Affiliations:** 1 School of Environmental Science, Nanjing Xiaozhuang University, Nanjing 211171, China; 2 Key Laboratory of Soil Environment and Pollution Remediation, Institute of Soil Science, Chinese Academy of Sciences, Nanjing 210008, China; sakura8586@163.com (X.W.); soil1619@163.com (Z.Z.); 3 Ecological Experimental Station of Red Soil, Chinese Academy of Sciences, Yingtan 335211, China

**Keywords:** small-scale pig farming, water pollution, P accumulation, heavy metal accumulation, crop safety

## Abstract

Industrialized small-scale pig farming has been rapidly developed in developing regions such as China and Southeast Asia, but the environmental problems accompanying pig farming have not been fully recognized. This study investigated 168 small-scale pig farms and 29 example pig farms in Yujiang County of China to examine current and potential impacts of pig wastes on soil, water and crop qualities in the hilly red soil region, China. The results indicated that the small-scale pig farms produced considerable annual yields of wastes, with medians of 216, 333 and 773 ton yr^−1^ per pig farm for manure, urine and washing wastewater, respectively, which has had significant impact on surface water quality. Taking NH_4_^+^-N, total nitrogen (TN) or total phosphorus (TP) as a criterion to judge water quality, the proportions of Class III and below Class III waters in the local surface waters were 66.2%, 78.7% and 72.5%. The well water (shallow groundwater) quality near these pig farms met the water quality standards by a wide margin. The annual output of pollutants from pig farms was the most important factor correlated with the nutrients and heavy metals in soils, and the relationship can be described by a linear equation. The impact on croplands was marked by the excessive accumulation of available phosphorus and heavy metals such as Cu and Zn. For crop safety, the over-limit ratio of Zn in vegetable samples reached 60%, other heavy metals in vegetable and rice samples tested met the food safety standard at present.

## 1. Introduction

In the past two decades there has been a rapid development of swine husbandry in China, with a sold hog amount of 716 million heads accounting for about 50% of the total swine production in the world [1]. China has the largest swine production in the world, but its swine industry is not equally strong yet in terms of productivity. The industrialization and concentration degree of pig farming in China still needs to be improved compared to developed countries, although the proportion of industrialized pig farms has been increased after several years of swine production structure adjustment [2]. At present, small-scale pig farming (defined hereafter as being less than 1000 heads in annual sold hog amount through industrialized feeding operations, not including traditional backyard pig feeding in rural areas) still dominates in the swine industry of China [3]. Not only for China, there is also a high swine production and relatively high pig densities in Southeast Asia, most of which is associated with smallholders’ pig farms operated on a small scale [4]. Taking Vietnam as an example, 80% of its pig population is kept in smallholders’ feedlots [5]. In these areas, most small-scale pig farms discharge pig wastes, mainly including pig manure, urine and washing wastewater, to the environment after preliminary treatments or even without any treatment, compared to pig farms in developed countries, which are equipped with waste processing facilities to dispose of pig wastes properly regardless of pig farm size [6].

Public concern about environmental pollution from intensive swine production is gradually increasing. Much of the concern focuses on nitrogen (N) and phosphorus (P) pollution of surface and groundwater, and heavy metal pollution of soils and plants [7], because pig waste is a significant source of N, P and heavy metals [8,9]. N and P are the two primary nutrients leading to eutrophication of lakes and streams, which can deteriorate water quality and affect the normal functioning of water bodies [10,11]. Studies have revealed that livestock and poultry farming are the main cause of water eutrophication compared to industrial pollution in China [12]. Moreover, high nitrate (NO_3_^−^) in groundwater resulted from pig farming is harmful to human health, and in particular, ingestion of nitrate may cause methemoglobinemia in infants [13,14]. Direct release of untreated pig wastes to surrounding croplands can lead to heavy metal enrichment in soil and then probably in crop, which may pose a potential health risk to humans [15]. Copper (Cu), zinc (Zn) and cadmium (Cd) pollution caused by pig manure application in agricultural soils have been widely reported [16,17]. Traditionally, the impact of small-scale pig farming on the environment is considered to be negligible as the environment around a small-scale pig farm has an adequate assimilatory capacity to digest the wastes produced from the pig farm. However, the self-cleaning ability of the environment can be limited in the long term as some pollutants may build up to a harmful level due to a long-term waste loading, although they are below environmental quality standards in the early stages of small-scale pig farming [18]. With regard to small-scale pig farming in China and other developing regions, lack of proper treatment facilities together with mismanagement of pig wastes may pose a threat to the environment quality [19,20]. It is imperative to examine the impacts of small-scale pig farming on the local environment, with a special attention to its potential risks in the long term.

The hilly red soil region is an important area for swine husbandry in subtropical and tropical southern China. Yujiang County, Jiangxi Province is a typical representative for small-scale pig farming in the region, with small-scale pig farms accounting for 43.2% of total pig farms in the county [6]. To evaluate current and potential impacts of small-scale pig farming on the environment, this study investigated 168 small-scale farms to acquire basic information on inception time, production capacity, waste disposal and so on, and then selected 29 typical small-scale pig farms as the sample farms for further investigation. In these selected small-scale pig farms in the county, we determined the outputs of wastes and main contaminants, qualities of local soil, water, agricultural produce, and the cumulative rates of heavy metals accumulation in soil. Based on this information, we assessed current and future environmental and health risks in the study area. This study will provide important information for policy makers to improve the regulations on small-scale pig farming in the region and other developing countries where small-scale pig farming prevails.

## 2. Materials and Methods

### 2.1. General Situation of Study Area

Yujiang County is located in the northeast of Jiangxi Province, China. Its total area is 937 km^2^ between 28°04′–28°37′ N and 116°41′–117°09′ E. This study area is characterized by a typical subtropical humid monsoon climate with an annual average temperature of 17.7 °C (a maximum daily temperature of approximately 40 °C in summer), an average of 269 frost-free days, and annual rainfall of 1754 mm, approximately 50% of which falls from March to June. There are three dominating upland soils in this region, Agri-Udic Ferrosols (Oxisols, US ST) from red clay, Ali-Udic Argosols (Ultisols, US ST) from sandstone and Ali-Udic Cambisols (Inceptisols, US ST) from granite soil. According to previous investigation, the bulk density, soil water content, pH and clay content are 1.14–1.55 g cm^−3^, 8.92–27.69%, 4.05–6.83% and 9.76–44.41% respectively. The values of cation exchange capacity (CEC), total organic carbon (SOC), total nitrogen and total phosphorus concentrations in the study area are 10.93–15.68 cmol kg^−1^, 3.87–45.93 g kg^−1^, 0.47–3.32 g kg^−1^ and 0.26–4.09 g kg^−1^, respectively.

The terrain of Yujiang County is higher in both its south and north parts, and then gradually inclines to its central region. The majority of the county is low-hilly land with an area of 720 km^2^, with a flourishing river system, water ponds and reservoirs distributed throughout the county, with a water area of 90 km^2^. The county has 600 km^2^ of arable land comprising 320 km^2^ of upland and 280 km^2^ of paddy field. These natural conditions are favorable for pig husbandry development in the county. Yujiang is one of the top ten counties in swine production in Jiangxi Province and its pig production accounts for over 80% of its total livestock production [21].

### 2.2. Research Methods

#### 2.2.1. Data and Sample Collection

**Figure 1:**
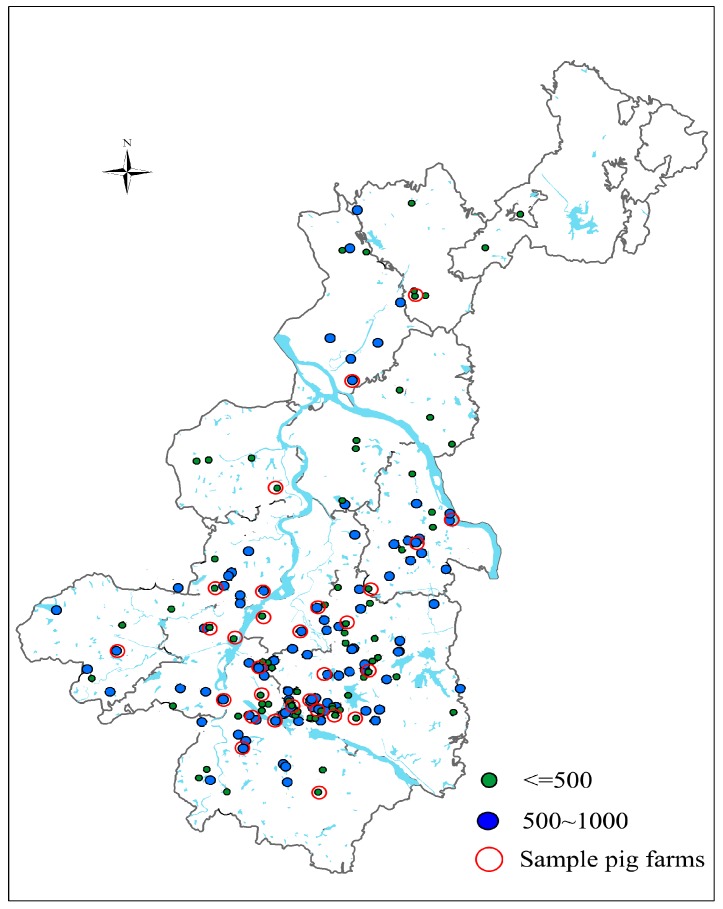
Distribution of small-scale pig farms (≤500 and 500–1000 heads in annual sold hog) and sample pig farms surveyed in Yujiang County, Jiangxi Province, China.

According to our previous study [6], there were 168 small-scale pig farms in Yujiang County, accounting for 43.2% of the total 389 industrialized pig farms in the county (Figure 1). The basic information of the small-scale pig farms on inception year, location (GPS value), production status, waste disposal, distance from village, and surrounding environmental components was collected through site investigations and interviews with pig farm workers or managers in October 2015. Based on these basic data, 29 representative pig farms were selected as sample pig farms for further examination of the outputs of wastes and contaminants, qualities of local soil, water and agricultural products, the cumulative rates of heavy metals in soil, and current and future environmental and health risks in the study area. For this purpose, a composite sample was collected from each of the 29 pig farms for manure, wastewater, vegetable soil (hereafter representing the soil cultivated for vegetable production), foliar vegetable (mainly cabbage, *Brassica oleracea* L.), paddy soil and rice grain. The soil samples were collected three times in all and the sampling time was November of 2015, November of 2016, October of 2017, respectively.

The composite pig manure samples were directly collected each from the manure storage pool of each pig farm. The composite wastewater samples were collected from two or three storage cesspits, which received urine and washing wastewater (resulting from washing feedlot floors after dry cleaning of pig feces) released intermittently from each pig farm, and functioned as a settling tank. A wastewater sample was actually the mixture of pig urine and washing wastewater in this study. Soil samples (0–15 cm) were collected after harvesting from adjoining croplands growing vegetables and paddy rice, and vegetable and rice samples were collected when harvesting. The vegetable and paddy fields were applied with pig manure annually. Meanwhile, control soil samples (CK) were also collected from nearby vegetable and paddy fields to which no pig manure was applied. Since surface water and groundwater chemistry varies between seasons, we collected water samples in March, June, September and December of 2016. In this year, annual rainfall and evaporation came close to the average, heavy rainfall concentrated mostly in spring and summer. Therefore, it has good representativeness. Surface water samples were taken from the waste-receiving surface water (fishponds, reservoirs, and streams) near pig farms according to the area and type of water bodies. For the reservoir and fishponds with a water area of more than 0.03 km^2^, 5–10 water samples were collected about 150 m from the sewage into the pond and 10 m from the embankment (50 cm in depth) in each of the 29 pig farms. Similarly, 3–5 water samples were collected for the reservoir and fishponds with a water area of less than 0.03 km^2^. For the stream, 3–5 water samples were collected about 5 m from the sewage into the stream and 10 m from the embankment (50 cm in depth) or along the middle of the stream. Three composite water samples were taken from the shallow pipe well as groundwater source located within a distance of 50 m away from a pig house. Actually, these sampled wells were drilled within the pig farms by the pig farm owners to collect water for the pigs and washing feedlot floors.

The fresh manure samples were oven-dried at a low temperature of 40 °C and then ground in a pulverizer. The weights of the manure samples before and after oven-drying were recorded and their water contents were calculated accordingly. The water and wastewater samples were transported to the laboratory within 24 h of collection and then stored in a refrigerator (4 °C) prior to analysis. After collecting soil samples, large pieces of plant material and animal residue were removed, and the soil samples were air-dried at room temperature, passed through a 2-mm sieve to analyze the available nitrogen and phosphorus, and then through a 0.149-mm sieve to analyze total N, P and heavy metals. After being oven-dried at 45 °C, rice samples husked and then ground by a pulverizer. Vegetable samples (mainly cabbage, *Brassica oleracea* L.) were washed with tap water first in order to remove the sand, and then rinsed 2–3 times with high pure water. After that vegetable samples were oven-dried at 65 °C and then ground using pulverizer.

#### 2.2.2. Sample Analysis

The manure and plant samples were digested using H_2_SO_4_-H_2_O_2_ and their total N (TN) and total P (TP) were determined by Semi-Micro Kjeldahl and molybdenum blue colorimetrical methods, respectively. Their total concentrations of heavy metals were determined by atomic absorption spectroscopy (AAS) after the manure and plant samples were digested by HNO_3_-HClO_4_ and HNO_3_-H_2_O_2_, respectively. The concentrations of TN, TP, NH_4_^+^-N and NO_3_^−^-N in water sample were analyzed using a Smartchem 200 Discrete Auto Analyzer (Smartchem 200, AMS, Rome, Italy). Chemical oxygen demand (COD) of water and wastewater samples was measured by potassium dichromate method and determined using COD detector (COD-571, Leici, Shanghai, China). And heavy metal concentration was determined by atomic absorption after they were digested by HNO_3_-HClO_4_. Soil TN was determined by the Semi-Micro Kjeldahl method after soil samples were oxidized by sulfuric acid (H_2_SO_4_). Soil TP was determined by molybdenum blue colorimetrical method after soil samples were oxidized using H_2_SO_4_-HClO_4_. Soil available N (AN) was analyzed by alkaline hydrolysis diffusion method, available P (AP) was extracted with 0.5 M NaHCO_3_ and determined by the molybdenum blue method. Soil heavy metal concentrations were determined by atomic absorption after soil samples were digested by HNO_3_-H_2_O_2_-HF in sealed pots [22,23]. Total N (TN) and total P (TP) of manure, plant and soil samples were analyzed using a kjeltec auto sampler system (Kjeltec 8400, FOSS, Copenhagen, Denmark) and an ultraviolet visible spectrophotometer (Alpha-1506, Puyuan, Shanghai, China) respectively. Heavy metals in the samples were analyzed using atomic absorption spectrometer (Solaar M6, Thermo, MA, USA). Quality control samples derived from the soil standard reference sample of national standard material (GBW07416) and biological ingredients standard (GBW10045) were used during the detection for the purpose of verifying the accuracy of analysis. High pure water was used as a blank sample in order to eliminate the errors resulting from the effect of the outside measurement. The relative standard deviation (RSD %) of soil and plant samples was less than 5% by using these methods. The methods had good accuracy and recovery rate, which was between 92% and 102.7% for each addition. For flame AAS, the limits of determination of Cu, Zn and Pb were 0.0025 μg mL^−1^, 0.0175 μg mL^−1^ and 0.0224 μg mL^−1^. For a graphite furnace atomizer method, the limit of detection of Cd was 0.0119 ng mL^−1^.

#### 2.2.3. Data Analysis

Pig manure and urine are the two initial wastes from pig farming. The typical calculation method for annual yields of pig manure, urine or washing wastewater is expressed as,

*Y_w_* = (*H* × *k*_1_ × 160) + (*S* × *k*_2_ × 365) (1)

where *Y_w_* is the annual output of pig manure (or urine, or washing wastewater), *H* is the annual sold hog amount (head), *S* is the amount of sows (head), *k*_1_ is the daily waste production coefficient of hogs, and *k*_2_ is the daily waste production coefficient of sows. The daily waste production coefficients of hog and sow for manure, urine, washing wastewater are quoted from our previous study [6]. The daily production coefficients for manure, urine, and washing-induced wastewater for hog are 2.0, 3.3, and 8.0 kg head^−1^ day^−1^, respectively, and the breeding cycle for hog is 160 days. These daily waste production coefficients for sow are 4.0, 5.0, and 10.0 kg head^−1^·day^−1^, respectively, and its breeding cycle is 365 days [6]. There may be some errors using the daily average excretes for the estimation of waste production. Because quantity of poultry, raising period and nutrient elements in faeces were a set of dynamic data, which were affected by the types of pigs, individual size and composition of feed. It was also related to environmental factor such as season, climate and management levels.

In practice, the urine and wastewater resulting from washing feedlot floors are not drained separately, but share the same drainage channels, and actually are mixed in the drainage channels or storage cesspits in these small-scale pig farms [6]. For this reason, we have modified the computation method for pollutant yields from small-scale pig farming as follows,

*Y_p_* = *Y_w_* × *C_i_* (2)

where *Y_p_* is the annual yield of a certain pollutant (N, P, Cu, Zn, Cd and Pb in this study), *Y_w_* is the annual output of pig manure or mixed-up wastewater (i.e., the sum of urine and washing wastewater amounts), and *C_i_* is the concentration of a certain pollutant in manure or mixed-up wastewater. We used the measured *C_i_* values by analysis of collected samples in this study to calculate the pollutant yields from these sample small-scale farms.

For estimating the average cumulative rate of heavy metal in soil near a pig farm, it was assumed that the starting time of the accumulation was the inception year of the pig farm and the heavy metal concentrations of the control soil were the initial heavy metal concentrations in the inception year. So the average heavy metal accumulation rate was calculated as the quotient of the difference in heavy metal concentration between manured and the control soils and the time span from the inception year to the year when the soil samples were collected.

#### 2.2.4. Statistical Analysis

The means (*n* = 3) and standard deviations (SD) of the primary pollutants in fresh manure, wastewater and the annual outputs of major pollutants in pig wastes were presented. The impact factors on soil and water environment were tested using a redundancy discrimination analysis (RDA) (CANOCO 4.5). The correlation between the soil heavy metals and the annual outputs of pollutants from pig farm was determined by Origin version 8 software (OriginLab, Austin, MA, USA).

## 3. Results and Discussion

### 3.1. General Information on Small-Scale Pig Farming

The general information on small-scale pig farming in the study area is presented in Table 1. Most of the small-scale pig farms were set up after 2005, which was consistent with the rapid development of swine industry of China in that period [2]. The pig production scale varied from less than one hundred to nearly one thousand sold hogs/per year, with a median of 500 heads. Since these pig farms propagated and raised piglets on their own, the relatively low average sold hog per sow (14 heads) indicated that the productivity level of these small-scale pig farms was low, compared with the local average of 17 heads or the high average of 25 heads in developed countries [6]. Nevertheless, small-scale pig farming contributed to 13.7% of the total annual sold hog in the county. A large proportion of small-scale pig farms (46.6%) were located within a distance of 500 m from villages, which is the shortest distance permit for seating a pig farm away from village according to the Environment Impact Assessment Law of China enacted in September 2003. Some small-scale pig farms were even located within villages because the villages had been expanding with economic development and then some of these pig farms came to be surrounded by newly built houses. On an annual basis, these small-scale farms each produced considerable amounts of manure, urine and washing wastewater, with medians of 216, 333 and 773 tons, respectively. Most of these pig farms adopted a dry-cleaning method to remove manure from the feedlots (i.e., removing the manure from feedlot floors with a shovel, not by water flushing), and some pig farms adjacent to ponds or reservoirs flushed pig manure by water into the waters to feed fish. A small portion of these pig farms use both methods (Table 1). The manure output from these pig farms was applied to croplands/orchards and fish-raising waters, either accounting for half of the total. By contrast, most of the wastewater (mixed-up of urine and washing wastewater) produced from these pig farms was discharged to surface waters. The lower fraction of pig wastewater for agricultural use compared to pig manure could be due to the difficulties in its storage and transportation as well as its low nutrient contents. Biogas digesters have not been widely adopted to dispose of pig wastes in these small-scale pig farms, probably due to easier disposal of the limited manure and wastewater yield from a small-scale pig farm compared to a large-scale pig farm, in which biogas digesters or other provisions are required for pig waste disposal [6].

### 3.2. Annual Output of Wastes and Major Pollutants from Sample Pig Farms

29 sample pig farms were used for evaluating impacts of small-scale pig farming on the local environment. The information on inception time, pig and waste production for these sample farms is listed in Table 2. These sample pig farms showed a little higher wastes production than the population pig farms, which was in agreement with higher swine production (annual sold hog) in these sample pig farms (Table 1 and Table 2). The concentrations of N, P and some heavy metals (Cu, Zn, Cd and Pb) in fresh manure and mixed-up of urine and washing wastewater were given in Table 3. Pig manure contained appreciable concentrations of eutrophication-relevant N and P. The concentrations of Cu and Zn were very high in pig manure samples, with an average of 40.8 and 262.4 mg kg^−1^ on the fresh weight basis. Many studies have reported that Cu and Zn are the major metal pollutants in pig manure [24]. Cu and Zn have a positive effect on pig growth and reproduction, thus frequently added as supplements at high levels to pig feeds [25]. Approximately 80–95% of Cu and Zn dietary supplements is excreted in manure [26,27]. Measurable concentrations of N, P, Cu and Zn were detected in wastewater samples.

Annual outputs of major pollutants including eutrophic nutrients (N and P) and heavy metals (Cu, Zn, Cd and Pb) from these sample small-scale pig farms were calculated based on Table 2 and Table 3 and presented in Table 4. The discharges of total N, P, Cu, and Zn from these sample pig farms were considerable, with mean values of 1.87 ton yr^−1^, 0.51 ton yr^−1^, 46.4 kg yr^−1^ and 297 kg yr^−1^, respectively. For small-scale pig farms, a large amount of wastes may be only treated with simple treatments or without any treatment and then directly discharged to the environment. The site investigation showed that pig manure was applied to croplands and orchards no more than 1 km away from the small-scale pig farm that produced the manure, and manure and/or wastewater were discharged to nearby receiving water bodies no more than 500 m away from the pig farm. In light of the limited dispersing distances for pig wastes, some pollutants could build up to a risky level in local environment around a small-scale pig farm.

### 3.3. Impacts of Small-Scale Pig Farming on Surface and Groundwater

Taking COD, NH_4_^+^-N, TN and TP as a criterion to judge water quality according to the surface water environmental quality standards of China (GB3838-2002) (COD < 20 mg L^−1^, NH_4_^+^-N < 1.0 mg L^−1^, TN < 1.0 mg L^−1^, TP < 0.2 mg L^−1^), the Class III and below Class III-water proportions of the local surface waters were 6.2%, 66.2%, 78.7% and 72.5% respectively. According to the European Union standard (COD < 15 mg L^−1^, NH_4_^+^-N < 0.8 mg L^−1^, TN < 8 mg L^−1^, TP < 0.4 mg L^−1^), the Class III and below Class III-water proportions of the local surface waters were 9.2%, 71.4%, 0% and 2.3% respectively. The concentrations of heavy metals in these surface waters were quite low and even not detectable, so one or two decades of small-scale pig farming has not led to heavy metal pollution of local surface waters. According to the investigation of small-scale pig farming in this region, the dry method of cleaning manure was widely used in 90% pig farms and contributed to about 64% of manure removal (Table 1), and pig manure was cleaned out every two or three days. Only the pig manure residue after dry cleaning was washed out by water and the resulting washing wastewater was drained to and mixed with pig urine in storage cesspits. The mixed-up wastewater in cesspits overflowed intermittently to the nearby water bodies. The waste effluent from small-scale pig farms might be the major source of water pollution. The quality of local surface waters near the small-scale pig farms also showed seasonal differences, and concentrations of COD, NH_4_^+^-N, NO_3_^−^-N, TN, TP, Cu and Zn all varied in the following order: winter (December) > autumn (September) > spring (March) > summer (June) (Table 5). This was largely the result of more rainfall in spring and summer, and dilution pollutants due to the increased water volume. In addition, aquatic organisms like phytoplankton may play an important role in purifying water in spring and summer, when their growth was favored [28]. On the other hand, the poorer water quality in autumn and winter could be attributed to the decreased water volume caused by less rainfall and reduced aquatic production due to low temperatures [29].

Nitrate was the main pollutant for groundwater, but the nitrate concentrations in the wells investigated through a year were much lower than the standard for drinking water in China (GB5749-2006, nitrate-N < 20 mg L^−1^). The concentrations were also lower than the European Union standard (nitrate-N < 11.3 mg L^−1^) and the American and Japanese standards (nitrate-N < 10 mg L^−1^). On the other hand, compared to the background values of nitrate concentration (0.16–1.35 mg L^−1^) in the groundwater in the study area [30], small-scale pig farming elevated the nitrate concentration to some extent in groundwater near the pig farms. Cu and Zn concentrations were very low or even not detectable in well water samples (Table 6), which were far below the Chinese standard (Cu < 1 mg L^−1^, Zn < 1 mg L^−1^) and American standards (Cu < 1.3 mg L^−1^, Zn < 5 mg L^−1^). Cd and Pb were not detected in groundwater samples (data not shown). These results indicate that the groundwater quality can meet the water quality standards at present. Actually the source of pollution in surface water and groundwater water sampling stations not only came from pig farms but also caused by the agricultural and residential pollution. Due to wide application of fertilizers and pesticides, agricultural non-point sources have been the key pollution source of water environment in China. Meanwhile the discharging of sewage and living garbage into the river had already greatly imperiled water environment security.

### 3.4. Impacts of Small-Scale Pig Farming on Soil Environment

Pig farming significantly increased concentrations of major nutrients in local soils, particularly for soil-available phosphorus (AP), ranging 121.2–349.6 mg kg^−1^ for vegetable soil and 120.4–373.2 mg kg^−1^ for paddy soil, with averages being more than four and six the control soils (CK), respectively (Table 7). The accumulation of N was much lower than that of P, probably because the N/P ratio of pig manure is often narrower than the N/P uptake ratio of most crops and N is more susceptible to be lost out of the soil than P [31,32]. The low mobility of P and the high adsorption of soil for P make it easily accumulate in soil, moreover, the utilization of phosphorus is only 10–25% for one current cropping, and others accumulate in the soil in different forms [33].

Heavy metal accumulation was pronounced in soils near the pig farms. Compared to CK, the average concentrations of Cu and Zn were nearly 3 times higher in vegetable soils. The soil samples investigated were all below the Class II soil quality standard in terms of Cu concentration according to the soil environmental quality standards of China (GB15618-1995). The probability of Zn meeting the Class II soil quality standard was 16.7%. But the contents of Cu and Zn were much higher than quality criteria of soil environment allows in America (Cu < 70 mg kg^−1^, Zn < 160 mg kg^−1^) and Holland (Cu < 36 mg kg^−1^, Zn < 140 mg kg^−1^). Other heavy metals (Cd and Pb) were not only within the scope of Class II soil quality standards at present, but also meeting the soil environmental quality standards of America (Cd < 4 mg kg^−1^, Pb < 120 mg kg^−1^) and Holland (Cd < 0.8 mg kg^−1^, Pb < 85 mg kg^−1^). To determine the potential risk of heavy metals in the soil environment, the annual cumulative rate of heavy metals was calculated (Table 8). Based on the Class II soil environmental quality standards (pH < 6.5, Cu < 50 mg kg^−1^, Zn < 200 mg kg^−1^, Cd < 0.3 mg kg^−1^, Pb < 250 mg kg^−1^), soil Zn concentration, which is still qualified at present, will exceed the quality standard in 2–4 years. For Cd, the concentration in vegetable soils will exceed the upper limit of quality standard in 4–22 years. The risk for Pb is negligible in the vegetable soils.

Although the average concentrations of Cu and Zn in paddy soils near these pig farms were approximately 2 times higher than the CK, the probabilities of Cu and Zn meeting the Class II soil environmental quality standards still reached 86.3% and 87.1%, respectively, mainly due to the lower background value (Table 7). The probability of Cd to meet the Class II soil quality was 91.7%, and the concentration of Pb was within the limit of Class II soil quality standards at present. According to the soil environmental quality standards of America, the probabilities of Cu, Zn, Cd and Pb were 96.6%, 71.3%, 100% and 100% respectively. The national environmental standard for Cu, Zn, Cd in Holland was stricter than in America, the probabilities reached to 67.8%, 64.4%, 97.7%. The concentration of every tested heavy metal in paddy soil was much lower than that in vegetable soil, due partially to higher manure application rates used in vegetable soil. According to the annual cumulative rate of heavy metal in paddy soil, the concentrations of Cu, Zn and Cd, which are still qualified at present, will reach the ceiling in 6–32, 5–33 and 5–20 years, respectively, if the paddy soils continue to be manured as usual. The risk for Pb accumulation would be insignificant in the paddy soil. 

### 3.5. Impacts of Small-Scale Pig Farming on Crop Safety

Crop safety has been a hot spot in recent research and attracted public attention. The heavy metal contents of Cu in Chinese cabbage (*Brassica oleracea* L.) meet the food quality standard for vegetables (GB15199-94, Cu < 10 mg kg^−1^) (Table 9), although excessive accumulation of Cu was seen in the vegetable soils. Previous studies also revealed that the higher concentration of heavy metals in soil would not necessarily result in significant accumulation in crops [34,35]. The average concentration of Zn was above the upper limit (20 mg kg^−1^ Zn) of the food quality standard for vegetables, and the over-limit ratio reached 60%. Heavy metal concentrations of Cu and Zn in rice all meet the food quality standard for cereal (Cu < 10 mg kg^−1^, Zn < 50 mg kg^−1^). On the basis of the common standards for contaminants and toxins in food and feed by Codex Alimentarius Commission (CODEX STAN193-1995, Cd < 0.2 mg kg^−1^, Pb < 0.3 mg kg^−1^), the concentrations of Cd and Pb in vegetables were all qualified.

### 3.6. Impact Factors on Soil and Water Environment

**Figure 2:**
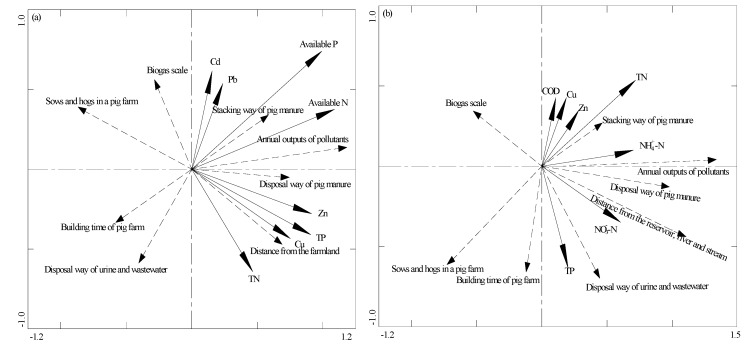
Redundancy discrimination analysis bi-plot showing the impact factors on soil environment (**a**) and water environment (**b**).

The RDA was used to further evaluate the impact factors on soil and water environment (Figure 2). The vector bio-plot showed that the selected impact factors (annual outputs of pollutants, stacking way of pig manure, distance from the farmland and disposal way of pig manure) were positively correlated with the soil heavy metals and nutrients contents. The strongest effect (longest vector length) on soil environment was annual outputs of pollutants. Regarding the soil nutrients and heavy metals, however, the selected impact factors, including sows and hogs in a pig farm, disposal methods of urine and wastewater, building time of pig farms and biogas scale, were not significantly correlated. For the water environment, the selected impact factors (annual outputs of pollutants, distance from the reservoir, stream and river, stacking way of pig manure, disposal way of urine and wastewater and disposal way of pig manure) were positively correlated with the nitrogen, phosphorus, COD, Cu and Zn. But the correlation was not significant for the impact factors such as sows and hogs in a pig farm, building time of pig farms and biogas scale. Among the selected impact factors, the annual outputs of pollutants from pig farms was the most important factors.

### 3.7. The Relationship between Outputs of Pollutants from Pig Farm and Soil Heavy Metals in the Vicinity of Sample Pig Farms

**Figure 3:**
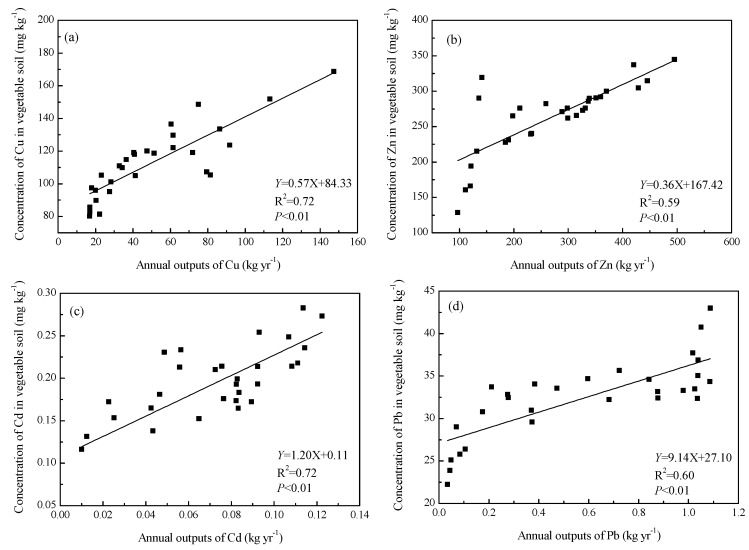
The heavy metals such as Cu, Zn, Cd and Pb in vegetable soil response to the annual outputs of pollutants from pig farms. (**a**) The relationship between annual outputs of Cu and content of Cu in vegetable soil. (**b**) The relationship between annual outputs of Zn and content of Zn in vegetable soil. (**c**) The relationship between annual outputs of Cd and content of Cd in vegetable soil. (**d**) The relationship between annual outputs of Pb and content of Pb in vegetable soil.

Because the strongest effect on soil environment was from the outputs of pollutants from pig farm, the relationship between the outputs of pollutants from pig farm and soil heavy metals was further investigated. Figure 3 showed the concenration of Cu, Zn, Cd and Pb in vegetable soils had linear correlations with the annual outputs of pollutants from pig farm.

**Figure 4:**
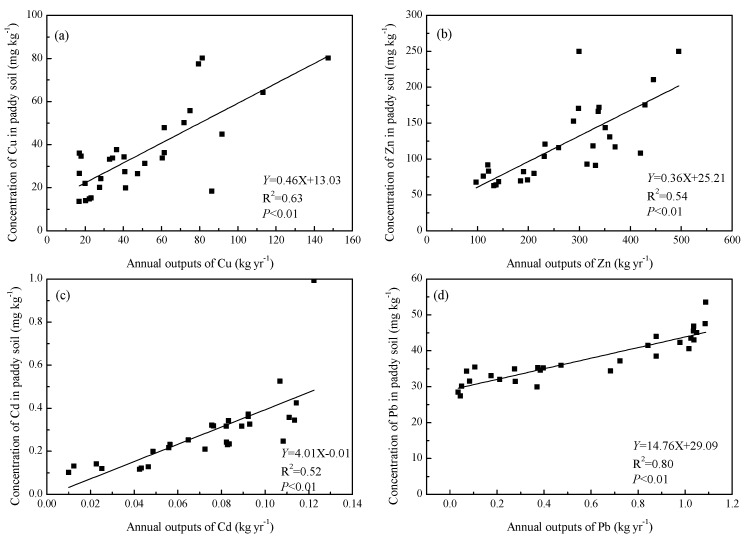
The heavy metals such as Cu, Zn, Cd and Pb in paddy soil response to the annual outputs of pollutants from pig farms. (**a**) The relationship between annual outputs of Cu and content of Cu in paddy soil. (**b**) The relationship between annual outputs of Zn and content of Zn in paddy soil. (**c**) The relationship between annual outputs of Cd and content of Cd in paddy soil. (**d**) The relationship between annual outputs of Pb and content of Pb in paddy soil.

The correlation coefficient (*p* < 0.01) was significant for Cu (*R*^2^ = 0.72), Zn (*R*^2^ = 0.59), Cd (*R*^2^ = 0.72) and Pb (*R*^2^ = 0.60). Relationship between the concenration of Cu, Zn, Cd and Pb in paddy soils and annual outputs of pollutants from pig farm was shown in Figure 4. The regression had a coefficient of 0.58 ** for Cu, 0.54 ** for Zn, 0.52 ** for Cd and 0.80 ** (** means the significance of regression coefficient, *p* < 0.01) for Pb, indicating a significant correlation between the indices. This indicated that it was necessary to control the discharge of pollutants from the source in order to reduce the accumulation of heavy metals in soil. It is therefore essential to strengthen planning in development of pig farming in the region, and intensify research on technologies for deharming and recycling of pig dung, urine and wastewater, to promote healthy development of small-scale pig farming and environment protection in red soil hilly areas.

### 3.8. The Relationship between Outputs of Pollutants from Pig Farm and Quality of the Waterenvironment in the Vicinity of Sample Pig Farms

**Figure 5:**
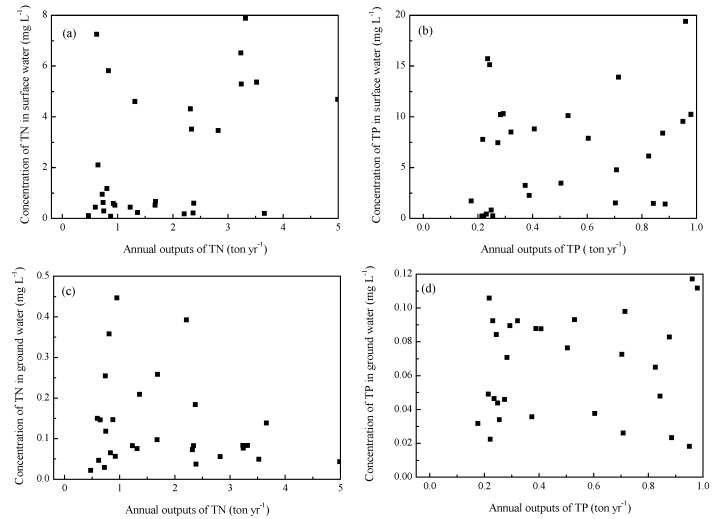
The concentrations of TN and TP in surface and ground water response to the annual outputs of pollutants from pig farms. (**a**) The relationship between annual outputs of TN and concentration of TN in surface water (**b**) The relationship between annual outputs of TP and concentration of TP in surface water (**c**) The relationship between annual outputs of TN and concentration of TN in ground water (**d**) The relationship between annual outputs of TP and concentration of TP in ground water.

Figure 2 shows the surface and ground water environments were significantly affected by the outputs of pollutants from pig farms. However, there was no obvious functional relationship between the outputs of TN, TP and concentrations in water environment, the relationship cannot be quantified (Figure 5). This was different from soil environment, which can be described by linear equation (Figure 3 and Figure 4). It was chiefly because heavy metals migrated slowly and its accumulation was pronounced in soils near the pig farms over a period of years. However, climate changes and seasonal rainfall both had significant effect on water environment, leading to a big random fluctuation of total nitrogen and phosphorus concentrations. Apart from potential contributions from other sources, it also involved mobilization, delivery by different pathways, transformation, and attenuation. Therefore, it is necessary to have long-term monitoring of the surface and ground water environment near the pig farms, in order to investigate the impact of outputs of pollutants from pig farms on water environment for further study.

## 4. Conclusions

The findings from this study indicated that solid and liquid wastes generated from small-scale pig farming were considerable, and could pose a great risk to the environment when they were discharged without effective pretreatments. Most of the waste-receiving surface waters (66.2–78.7%) were below the Class III water quality standard in terms of NH_4_^+^-N, TN or TP concentration. Excessive accumulation of available phosphorus, total Cu and Zn occurred in manured croplands near the small-scale pig farms and Zn enrichment was observed in vegetable samples. The annual outputs of pollutants from pig farms was the most important factor correlated with the nutrients and heavy metals in soils and water environment. The relationship can be described by a significant linear correlation between the indices in soils. But there was no significant functional relationship between the outputs of TN, TP and concentrations in water environment. This study can assist in understanding the environmental and health risks associated with small-scale pig farming in developing regions, and underline the need for better management of small-scale pig farms to reduce water, soil and crop pollutions.

## Figures and Tables

**Table 1:** Basic information on small-scale pig farming in the study area (*n* = 168).

Content	Quantity
Proportion of pig farms starting	
before 2001	15.30%
in 2001–2005	28.50%
after 2005 (until 2015)	56.20%
Sows in a pig farm (head)	
range	7–104
median	40
Annual sold hogs from a pig farm (head yr^−1^)	
range	70–980
median	500
Average sold hog amount per sow (head)	14
Contribution of annual sold hog from small-scale pig farms to the total annual of the study county	13.70%
Proportion of pig farms at a critical distance from village <500 m	46.60%
Annual manure production (ton yr^−1^)	
range	52–422
median	216
Annual urine production (ton yr^−1^)	
range	81–652
median	333
Annual washing wastewater production (ton yr^−1^)	
range	177–1524
median	773
Proportion of manure removal by	
dry-cleaning	63.70%
water flushing	28.10%
both	8.20%
Proportion of manure disposal by	
application to croplands and orchards	50%
application to fish-raising waters	50%
Proportion of wastewater disposal by	
discharge to water bodies	65%
irrigating croplands and orchards	35%
Percentage of pig farms mounted with biogas tank	13.60%

**Table 2:** Basic information on sample pig farms (*n* = 29).

Basic Information	Annual Rainfall (mm)	Annual Runoff (mm)	Inception Year	Sows in Stock (Head)	Annual Sold Hog (Head)	Fresh Manure (ton yr^−1^)	Urine (ton yr^−1^)	Washing Wastewater (ton yr^−1^)
Range	1472–1795	873–1302	1992–2009	25–60	300–990	137–404	210–632	486–1486
median	1553	1004	2003	56	700	306	472	1100

**Table 3:** Concentrations of primary pollutants in fresh manure and wastewater (mixed-up urine and washing wastewater) from sample pig farms (*n* = 29).

**Pollutants Source**	**Pollutants**	**TN (g kg^−1^)**	**TP (g kg^−1^)**	**Cu (mg kg^−1^)**	**Zn (mg kg^−1^)**	**Cd (mg kg^−1^)**	**Pb (mg kg^−1^)**
Manure	Range	3.7–11.7	2.7–7.0	19.4–110.0	121.6–331.5	0.017–0.119	0.14–1.01
	Mean	7.3	4.6	40.8	262.4	0.064	0.55
	Standard deviation	2.44	1.55	26.8	48.5	0.034	0.33
**Pollutants Source**	**Pollutants**	**TN (mg L^−1^)**	**TP (mg L^−1^)**	**Cu (mg L^−1^)**	**Zn (mg L^−1^)**	**Cd (mg L^−1^)**	**Pb (mg L^−1^)**
Wastewater	Range	0.25–1.69	0.004–0.031	0.02–0.29	0.03–5.81	0.0002–0.005	0.001–0.007
	Mean	0.79	0.012	0.13	0.97	0.0004	0.006
	Standard deviation	0.34	0.011	0.09	1.22	0.0008	0.001

**Table 4:** Annual outputs of major pollutants in pig wastes discharged from sample pig farms.

Pollutants	TN (ton yr^−1^)	TP (ton yr^−1^)	Cu (kg yr^−1^)	Zn (kg yr^−1^)	Cd (kg yr^−1^)	Pb (kg yr^−1^)
Range	0.47–3.66	0.22–0.94	16.99–147.4	97.8–495	0.01–0.11	0.08–1.09
Mean	1.87	0.51	46.4	297	0.07	0.64
Standard deviation	0.44	0.21	28.7	36.9	0.03	0.29

**Table 5:** Quality of the surface waters in the vicinity of sample pig farms.

Time	Treatment		COD	NH_4_^+^-N	NO_3_^−^-N	TN	TP	Cu	Zn
			(mg L^−1^)						
March	CK	Range	0	0.05–0.23	0–0.23	0.04–1.08	0	ND	ND
		Mean	0	0.14	0.12	0.67	0	ND	ND
	T	Range	0	0.35–0.63	0.28–0.58	0.47–3.48	0.02–0.14	ND	ND–0.021
		Mean	0	0.49	0.40	1.70	0.08	ND	0.02
June	CK	Range	0	0–0.24	0–0.11	0.01–0.63	0	ND	ND
		Mean	0	0.11	0.07	0.52	0	ND	ND
	T	Range	0	0–0.64	0–0.37	0.11–1.33	0.01–0.03	ND–0.002	ND–0.023
		Mean	0	0.26	0.18	0.82	0.02	0.001	0.016
September	CK	Range	0–4	0–0.89	0.08–0.23	0.36–1.05	0.01–0.04	ND	ND
		Mean	2	0.37	0.15	0.74	0.02	ND	ND
	T	Range	2–14	0.04–2.50	0.31–0.44	0.60–7.85	0.07–0.26	0.004–0.008	ND–0.10
		Mean	8	0.99	0.40	2.62	0.14	0.006	0.05
December	CK	Range	3–18	0.07–1.03	0.11–0.26	0.51–0.92	0.02–0.11	ND	ND
		Mean	9	0.56	0.18	0.79	0.07	ND	ND
	T	Range	13–65	0.20–5.14	0.31–0.76	1.51–4.31	0.06–0.43	0.008–0.012	ND–0.13
		Mean	39	1.72	0.57	2.91	0.17	0.01	0.09

ND: not detected.

**Table 6:** Quality of the groundwater in the vicinity of sample pig farms.

Time	Treatment		COD	NH_4_^+^-N	NO_3_^−^-N	TN	TP	Cu	Zn
			(mg L^−1^)						
March	CK	Range	0	0–0.38	0.12–1.08	0.21–1.53	0	ND	ND
		Mean	0	0.12	0.66	0.77	0	ND	ND
	T	Range	0	0.21–0.58	0.27–5.82	0.21–6.81	0.03–0.06	ND	0–0.01
		Mean	0	0.40	3.05	3.51	0.04	ND	0.005
June	CK	Range	0	0–0.44	0.37–2.08	0.12–2.41	0	ND	ND
		Mean	0	0.16	1.25	1.12	0	ND	ND
	T	Range	0	0.09–1.33	2.40–18.2	1.62–19.3	0.07–0.11	ND	ND
		Mean	0	0.51	8.47	8.36	0.09	ND	ND
September	CK	Range	0	0.04–0.92	0.09–0.94	0.23–8.79	0.01–0.03	ND	ND
		Mean	0	0.28	0.72	1.35	0.02	ND	ND
	T	Range	0	0.08–2.09	0.63–6.89	1.10–11.9	0.03–0.05	ND–0.008	0.007–0.013
		Mean	0	0.76	4.37	7.91	0.04	0.006	0.009
December	CK	Range	0	0–0.04	0.22–1.64	0.09–3.25	0	ND	ND
		Mean	0	0.01	0.93	1.46	0	ND	ND
	T	Range	0	0–0.15	3.56–9.49	3.83–7.74	0.03–0.06	0.004–0.008	0.008–0.042
		Mean	0	0.06	5.78	5.44	0.04	0.005	0.019

ND: not detected.

**Table 7:** Contents of nutrients and heavy metals in soils in the vicinity of sample pig farms.

Crop	Treatment		TN (g kg^−1^)	TP (g kg^−1^)	AN (mg kg^−1^)	AP (mg kg^−1^)	Cu (mg kg^−1^)	Zn (mg kg^−1^)	Cd (mg kg^−1^)	Pb (mg kg^−1^)
Vegetable	CK	Range	0.78–1.25	0.61–0.68	115.3–157.7	33.2–85.9	16.5–36.5	82.1–90.3	0.07–0.12	21.7–22.8
		Mean	1.03	0.66	136.7	58.5	28.5	88.3	0.10	22.3
	T	Range	1.79–2.85	1.05–3.91	209.7–318.9	121.2–349.6	80.2–168.7	166.0–344.5	0.12–0.27	22.3–43.0
		Mean	1.92	1.27	241.9	244.7	112.9	253.4	0.17	31.6
	Grade II of soil heavy metal ①						0	16.7%	100%	100%
	Standards of soil heavy metal in America						0	0	100%	100%
	Standards of soil heavy metal in Holland						0	0	100%	100%
Paddy	CK	Range	0.78–1.74	0.42–0.61	117.7–165.3	18.4–56.2	12.2–26.5	27.5–72.4	0.01–0.18	2.9–8.5
		Mean	1.46	0.52	136.5	39.5	20.2	48.3	0.07	4.2
	T	Range	1.25–3.15	0.33–2.91	115.0–307.2	120.4–373.2	13.6–80.2	67.7–249.8	0.11–0.99	28.5–53.5
		Mean	2.14	1.15	261.3	253.6	33.5	102.3	0.22	35.9
	Grade II of soil heavy metal ①						86.3%	87.1%	91.7%	100%
	Standards of soil heavy metal in America						96.6%	71.3%	100%	100%
	Standards of soil heavy metal in Holland						67.8%	64.4%	97.7%	100%

① According to the soil environmental quality standards of China (GB15618-1995).

**Table 8:** Average cumulative rate of heavy metals in croplands in the vicinity of sample pig farms.

Crop		Cu (mg kg^−1^ yr^−1^)	Zn (mg kg^−1^ yr^−1^)	Cd (mg kg^−1^ yr^−1^)	Pb (mg kg^−1^ yr^−1^)
Vegetable	Range Mean	4.37–9.94 6.15	5.41–18.16 12.80	0.007–0.015 0.008	0.34–2.01 1.02
Paddy	Range Mean	1.01–3.13 1.79	4.05–6.18 5.00	0.004–0.014 0.007	0.38–1.21 0.84

**Table 9:** Contents of heavy metals in vegetables and rice grown in soils in the vicinity of sample pig farms.

Crop		TN (g kg^−1^)	TP (g kg^−1^)	Cu (mg kg^−1^)	Zn (mg kg^−1^)	Cd (mg kg^−1^)	Pb (mg kg^−1^)
Vegetable	Range	3.76–3.84	0.40–0.57	0.38–0.64	5.92–66.23	0.01–0.10	0.04–0.14
	Mean	3.77	0.47	0.49	33.15	0.05	0.08
Paddy	Range	0.85–1.70	0.27–0.40	4.08–8.96	25.14–38.74	ND	ND
	Mean	1.18	0.33	6.74	27.81	ND	ND

ND: not detected. The concentrations of heavy metals in vegetable were calculated on a fresh weight base, and water content of vegetable was 92% on average.
